# Obesity reduces left ventricular strains, torsion, and synchrony in mouse models: a cine displacement encoding with stimulated echoes (DENSE) cardiovascular magnetic resonance study

**DOI:** 10.1186/1532-429X-15-109

**Published:** 2013-12-31

**Authors:** Sage P Kramer, David K Powell, Christopher M Haggerty, Cassi M Binkley, Andrea C Mattingly, Lisa A Cassis, Frederick H Epstein, Brandon K Fornwalt

**Affiliations:** 1 Departments of Pediatrics, Physiology and Medicine, University of Kentucky, 800 Rose St, MN-150, Lexington, KY 40536, USA; 2 Graduate Center for Biomedical Engineering, University of Kentucky, Lexington, KY, USA; 3 Department of Molecular and Biomedical Pharmacology, University of Kentucky, Lexington, KY, USA; 4 Departments of Biomedical Engineering and Radiology, University of Virginia, Charlottesville, VA, USA

**Keywords:** Obesity, Mouse, Cardiovascular magnetic resonance, DENSE, Strain, Heart

## Abstract

**Background:**

Obesity affects a third of adults in the US and results in an increased risk of cardiovascular mortality. While the mechanisms underlying this increased risk are not well understood, animal models of obesity have shown direct effects on the heart such as steatosis and fibrosis, which may affect cardiac function. However, the effect of obesity on cardiac function in animal models is not well-defined. We hypothesized that diet-induced obesity in mice reduces strain, torsion, and synchrony in the left ventricle (LV).

**Methods:**

Ten 12-week-old C57BL/6 J mice were randomized to a high-fat or low-fat diet. After 5 months on the diet, mice were imaged with a 7 T ClinScan using a cine DENSE protocol. Three short-axis and two long-axis slices were acquired for quantification of strains, torsion and synchrony in the left ventricle.

**Results:**

Left ventricular mass was increased by 15% (p = 0.032) with no change in volumes or ejection fraction. Subepicardial strain was lower in the obese mice with a 40% reduction in circumferential strain (p = 0.008) a 53% reduction in radial strain (p = 0.032) and a trend towards a 19% reduction in longitudinal strain (p = 0.056). By contrast, subendocardial strain was modestly reduced in the obese mice in the circumferential direction by 12% (p = 0.028), and no different in the radial (p = 0.690) or longitudinal (p = 0.602) directions. Peak torsion was reduced by 34% (p = 0.028). Synchrony of contraction was also reduced (p = 0.032) with a time delay in the septal-to-lateral direction.

**Conclusions:**

Diet-induced obesity reduces left ventricular strains and torsion in mice. Reductions in cardiac strain are mostly limited to the subepicardium, with relative preservation of function in the subendocardium. Diet-induced obesity also leads to reduced synchrony of contraction and hypertrophy in mouse models.

## Background

Obesity affects one third of adults [1] and one in five children [2] in the United States and is associated with increased mortality [3]. The increased mortality is primarily due to cardiovascular disease [4], but cannot be entirely explained by traditional risk factors such as diabetes, hypertension and dyslipidemia [5]. Animal (primarily rodent) models have shown lipid deposition [6], fibrosis [7] and altered myofilament proteins [8] in the heart as a result of obesity. These changes may affect cardiac function and be at least partially responsible for the increased cardiovascular mortality associated with obesity. However, the effect of obesity on cardiac function in animal models is *not* well-defined.

For example, some studies have shown cardiac dysfunction in mouse models of obesity [9-16], while others have shown no dysfunction [17-24]. These studies only utilized routine measures of cardiac function such as ejection fraction and ventricular dimensions. However, growing evidence shows that advanced measures of cardiac function (strain, torsion, and synchrony) are superior predictors of outcomes in patients with cardiovascular disease [25,26]. Preliminary human studies focusing on these advanced measures show that patients with obesity have *normal* ejection fraction and ventricular dimensions with *reduced* cardiac strains and torsion [27,28]. No study has quantified advanced cardiac function in a mouse model of diet-induced obesity. It is important to overcome this limitation to 1) establish relevance of the mouse model to human disease in order to investigate therapies and mechanisms and 2) further our understanding of the important link between obesity and cardiovascular mortality.

We aimed to overcome these limitations by using a Cardiovascular Magnetic Resonance (CMR) protocol called Displacement Encoding with Stimulated Echoes (DENSE) [29] to quantify cardiac function in mice with diet-induced obesity. Cine DENSE imaging is ideally suited for this problem due to its high spatial resolution, relatively quick and reproducible post-processing techniques, and proven accuracy for quantifying advanced measures of cardiac function [30]. We hypothesized that diet-induced obesity in mice would lead to a decrease in strain, torsion, and synchrony of contraction in the left ventricle.

## Methods

### Diet-induced obese and control mice

Ten 12-week-old C57BL/6 J mice were randomized to either a high fat diet *ad libitum*, with 60% of calories from fat (Research Diets #D12492) or a low fat diet with 10% of calories from fat (Research Diets #D12450B). Animals were housed in ventilated cages in a temperature-controlled room with a 14:10 light:dark cycle. Body weights were quantified weekly. All animal procedures conformed to Public Health Service policies for humane care and use of animals, and all procedures were approved by the Institutional Animal Care and Use Committee (IACUC) at the University of Kentucky.

### Animal preparation

Imaging was performed 5 months after starting the diet. Animals were anesthetized with 1.5-2.5% isoflurane in oxygen at 1.0 L/min. Isoflurane was adjusted to maintain respiratory rates of 100–140 breaths per minute. Three legs were shaved for placement of cutaneous ECG electrodes for cardiac gating, and the mouse was transferred to the scanning bed. A diaphragm to sense breathing was placed under the abdomen for respiratory gating. Core temperature was maintained from 36-37°C with a heated water blanket and rectal thermometer. All vital signs were continuously monitored with a fiber optic system (SA Instruments, Inc, Stony Brook, NY).

### CMR

**Figure 1:**
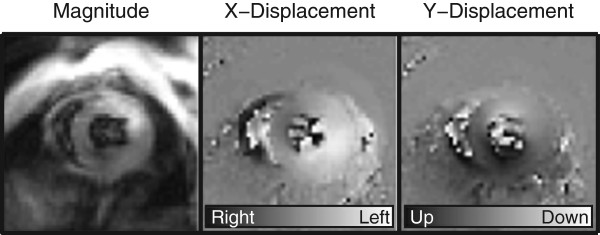
**Cine DENSE CMR images of a mouse heart collected on the Bruker 7 Tesla ClinScan.** Cine DENSE is an advanced magnetic resonance imaging technique, which can be used to show the location of the heart (magnitude image) and the phase-encoded × and y displacements. Pixel values in the phase-encoded displacement images represent the amount of displacement which has occurred between the current frame and the end-diastolic frame (see legends at bottom of × and y displacement images). This enables direct pixel-wise calculation of strains, torsion and dyssynchrony to quantify left ventricular function.

CMR was performed on a 7-Tesla Bruker ClinScan (Bruker, Ettlingen, Germany) with a 4-element phased array cardiac coil and a gradient system with a maximum strength of 450 mT/m and a slew rate of 4500 mT/m/s. Image acquisition has been described in detail previously [31,32]. Briefly, the CMR tissue tracking method known as cine *Displacement Encoding* with *Stimulated Echoes* (DENSE) was utilized. Immediately after an electrocardiogram R-wave trigger detection, which marks the depolarization of the ventricles and onset of contraction, a displacement encoding module consisting of radiofrequency and gradient pulses was applied, which stores position-encoded magnetization. This was followed by successive applications of a readout module which employed a radiofrequency excitation pulse, a displacement un-encoding gradient, and an interleaved spiral k-space trajectory. Ultimately, the output of the cine DENSE sequence was 3 images: a magnitude image showing the location of the heart and two phase images showing the horizontal X-displacement and vertical Y-displacement of each pixel (Figure 1). Relevant acquisition parameters included: field of view = 32 mm, matrix = 128 × 128, slice thickness = 1 mm, repetition time = 7.1 ms (15–20 frames per cardiac cycle), echo time = 0.67 ms, averages = 3, spiral interleaves = 36, and displacement encoding frequency = 0.8-1.0 cycles/mm. Each two-dimensional image acquisition took approximately 6–9 minutes depending on the heart and respiratory rates.

### Image slice selection

We acquired 3 short-axis and 2 long-axis images for each mouse. The long-axis images consisted of a standard apical 4-chamber view and a 2-chamber view perpendicular to the 4-chamber view. The short-axis images were planned perpendicular to the 4-chamber long-axis end-systolic image at the basal, apical and mid-ventricular locations. The mid-ventricular location was prescribed 50% of the end-systolic long-axis ventricular length above the apex while the basal and apical slices were prescribed at a distance 20% of the end-systolic length above and below the mid-ventricle.

### Image analysis

**Figure 2:**
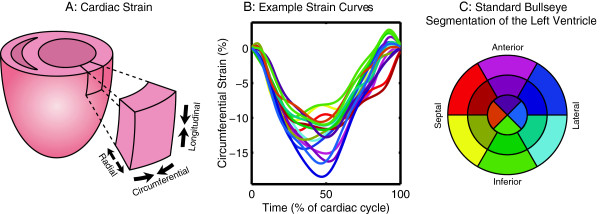
**Strain occurs in three primary directions in the left ventricle. (A)** Strain occurs in three primary directions, and is quantified as the percent change in length in the radial, circumferential and longitudinal directions relative to the end-diastolic (most relaxed) length. **(B)** Example strain curves from a control mouse are shown for the 16 standardized segments of the left ventricle, color-coded according to the bullseye in **(C)**. The peak strains occur as each segment reaches a minimum (i.e., at the point of maximum shortening). **(C)** Standard bullseye segmentation for the left ventricle includes dividing the ventricle into 3 slices: a basal slice further divided into 6 segments, a mid-ventricular slice further divided into 6 segments and an apical slice divided into 4 segments.

The displacement-encoded phase images were used to calculate strains, torsion and synchrony offline using custom software written in MATLAB (Mathworks, Inc., Natick, MA). Analysis included semi-automated motion-guided segmentation of the myocardium, phase unwrapping, tissue tracking to derive the path of each pixel throughout the cardiac cycle, and subsequent calculation of left ventricular strains [30,33]. The strains were used to quantify left ventricular synchrony using the circumferential and radial uniformity ratio estimate (CURE and RURE) indices [34]. Torsion was defined as the difference in twist angle between the basal and apical slices normalized by the long-axis epicardial length of the left ventricle at end-diastole (average of length measured on the 2-chamber and 4-chamber images) [35]. Global cardiac circumferential and radial strain curves were derived from averaging the strain curves of each of the 16 standardized segments of the left ventricle derived from the short-axis slices (Figure 2) [36].

Using methodology identical to that described above for the short-axis data, the long-axis data (from the two and four-chamber images) were used to calculate longitudinal strains. The apical segments were excluded from long-axis analysis since it is difficult to define longitudinal strain where the shape of the ventricle curves at the apex. Average longitudinal strains were calculated from both the two and four-chamber images. End-diastolic and end-systolic volumes, mass and ejection fraction were derived from a smooth, 3-dimensional reconstruction of the endocardial and epicardial borders defined from the DENSE magnitude images [37].

### Statistics

Analyses were performed in SPSS (IBM, Inc., Armonk, NY). Group differences were evaluated with a Mann–Whitney *U*-test. A value of p < 0.05 was defined as statistical significance. Values are reported as means ± SD.

## Results

**Figure 3:**
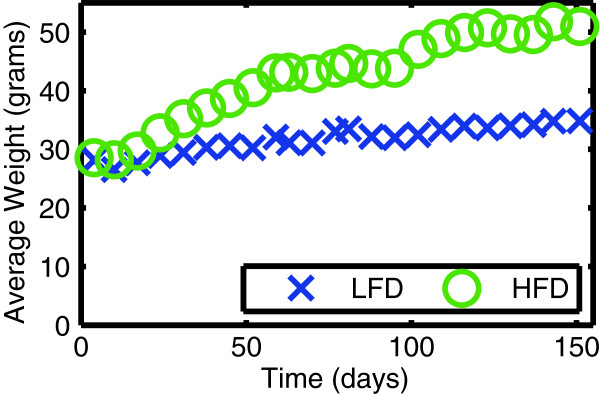
**Mice on a high-fat diet (HFD) develop obesity compared to control mice on a low-fat diet (LFD).** Weights are shown as a function of time. Diets were initiated at day 0 (at 12 weeks of age) and imaging was performed at 5 months.

The mice on the high-fat diet gained weight over the course of the 5 months (Figure 3).

### Left ventricular mass, volumes and ejection fraction

The obese mice had a 15% increase in left ventricular mass compared to the control mice (Table 1). Ventricular volumes were not significantly different. Ejection fraction trended towards being *increased* in the obese mice but was not significant (p = 0.056). The ratio of left ventricular mass to end diastolic volume was significantly increased in the obese mice (p = 0.032), consistent with a concentric hypertrophy.

**Table 1:** Obesity increases left ventricular mass with preservation of volumes and ejection fractions in mice

	**Low-fat diet**	**High-fat diet**	**Change due to high-fat diet**	**p value**
Left ventricular mass (mg)	100 ± 8	115 ± 8	+15%	0.032
Left ventricular end-diastolic volume (μL)	61 ± 8	58 ± 5	−6%	0.548
Left ventricular end-systolic volume (μL)	25 ± 5	21 ± 3	−16%	0.222
Left ventricular mass to end-diastolic volume ratio (mg/μL)	1.6 ± 0.3	2 ± 0.2	+22%	0.032
Left ventricular ejection fraction (%)	59 ± 3	64 ± 3	+7%	0.056

Values are mean ± standard deviation, n = 5 in each group.

### Left ventricular strains

**Figure 4:**
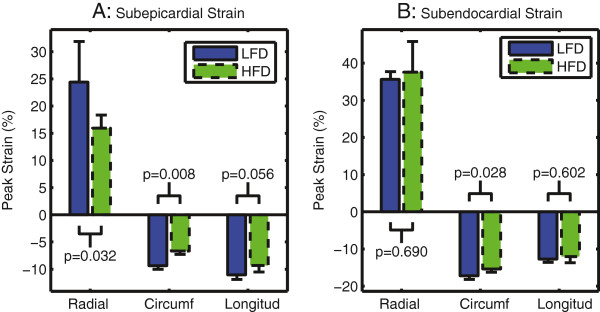
**Left ventricular strain is reduced in the subepicardium (A) with relative preservation of function in the subendocardium (B) in obese mice fed a high-fat diet (HFD) compared to mice on a low-fat diet (LFD).** Note that radial strain is positive because the heart thickens (lengthens) in this direction during systolic contraction while circumferential (circumf) and longitudinal (longitud) strains are negative because the heart shortens in these directions during systole. Bars show mean ± standard deviation.

**Figure 5:**
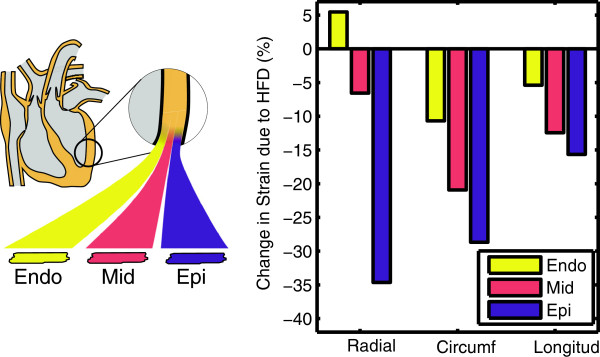
**Obesity has a differential effect on the strains in the myocardial layers of the left ventricle.** The cardiac wall can be subdivided into three layers (Endo = subendocardium, Mid = mid-myocardium, Epi = subepicardium) which are known to have different peak strains in normal mice. Left ventricular circumferential (circumf), radial, and longitudinal (longitud) strains are reduced in the subepicardium with relative preservation of function in the subendocardium in obese mice fed a high-fat diet (HFD). As an example, the purple bar for the reduction in subepicardial strain in the circumferential direction is equal to the difference in height of the green minus the blue bar in Figure 4A, expressed as a percentage of the blue bar.

Left ventricular subepicardial strain was reduced in the obese mice while subendocardial strain was relatively preserved (Table 2). The obese mice had a 40% reduction in the subepicardial circumferential strain (p = 0.008) and a 53% reduction in subepicardial radial strain (p = 0.032) (Figure 4). Subepicardial longitudinal strain trended towards a 19% reduction in the obese mice but was not significant (p = 0.056). By contrast, the obese mice had a modest 12% reduction in subendocardial circumferential strain (p = 0.028), and no significant change in the subendocardial radial (p = 0.690) or longitudinal (p = 0.602) strains. The differential effect of obesity on strain throughout the myocardial layers is depicted in Figure 5 showing that the greatest difference occurred in the subepicardium, with effects in the mid-myocardium and subendocardium being progressively smaller. Heart rate during the CMR was not significantly different between the two groups (Table 2). The average circumferential and radial strain were correlated with the mass to end-diastolic volume ratio (Table 3).

**Table 2:** Obesity reduces left ventricular strains, torsion and synchrony in mice

	**Region**	**Low-fat diet**	**High-fat diet**	**Change due to high-fat diet**	**p value**
Radial strain (%)	Endo	36 ± 2	38 ± 8	+5%	0.690
	Epi	24 ± 7	16 ± 2	−53%	0.032
Circumferential strain (%)	Endo	17 ± 1	15 ± 1	−12%	0.028
	Epi	9.4 ± 0.6	6.7 ± 0.6	−40%	0.008
Longitudinal strain (%)	Endo	13 ± 1	12 ± 2	−6%	0.602
	Epi	11 ± 0.8	9.3 ± 1.2	−19%	0.056
Peak torsion (^o^/cm)	N/A	8.8 ± 1.9	6.6 ± 0.5	−34%	0.028
Radial uniformity of strain (dimensionless)	Average	0.95 ± 0.02	0.91 ± 0.03	−5%	0.032
Circumferential uniformity of strain (dimensionless)	Average	0.99 ± 0.01	0.98 ± 0.01	−1%	0.151
Heart rate (bpm)	N/A	457 ± 35	503 ± 38	+10%	0.08

Values are mean ± standard deviation, n = 5 in each group. Endo = subendocardium, Epi = subepicardium.

**Table 3:** The left ventricular mass to end-diastolic volume ratio correlates with average circumferential and radial strain in the control and obese mice

	**R**	**p value**
Average circumferential strain	0.81	0.005
Average radial strain	−0.68	0.03
Average longitudinal strain	0.47	0.17

Correlations are reported for all 10 mice (5 on a low-fat diet and 5 on a high-fat diet).

### Left ventricular torsion

**Figure 6:**
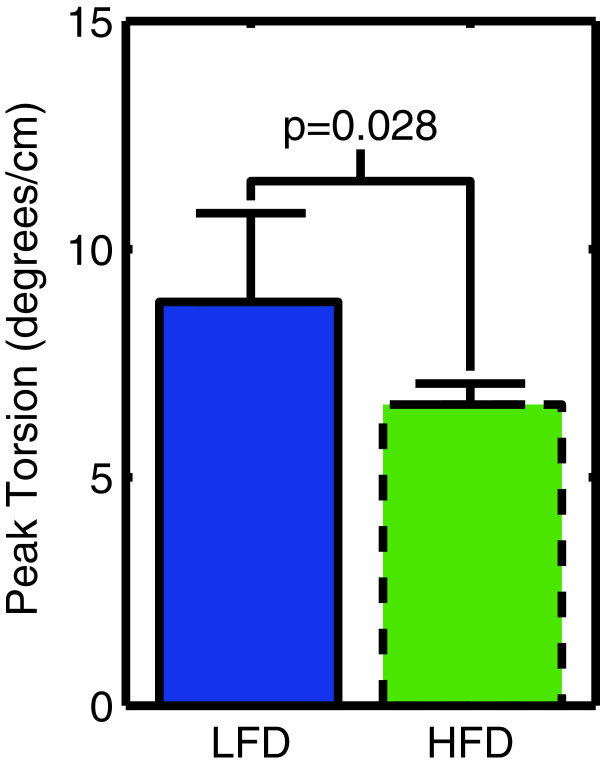
Left ventricular torsion is lower in obese mice fed a high-fat diet (HFD) compared to control mice on a low-fat diet (LFD).

Peak left ventricular torsion was reduced by 34% (p = 0.028) in the obese mice (Figure 6).

### Left ventricular synchrony

**Figure 7:**
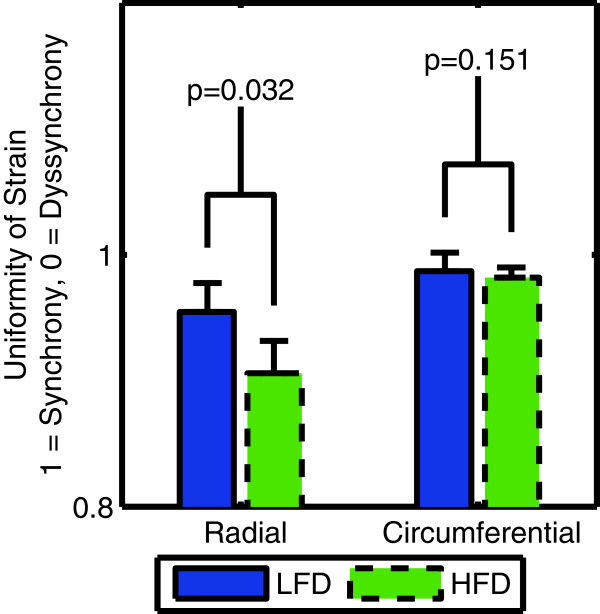
The radial uniformity of strain index shows a reduction in the synchrony of contraction in the left ventricle in obese mice fed a high-fat diet (HFD) compared to control mice on a low-fat diet (LFD).

**Figure 8:**
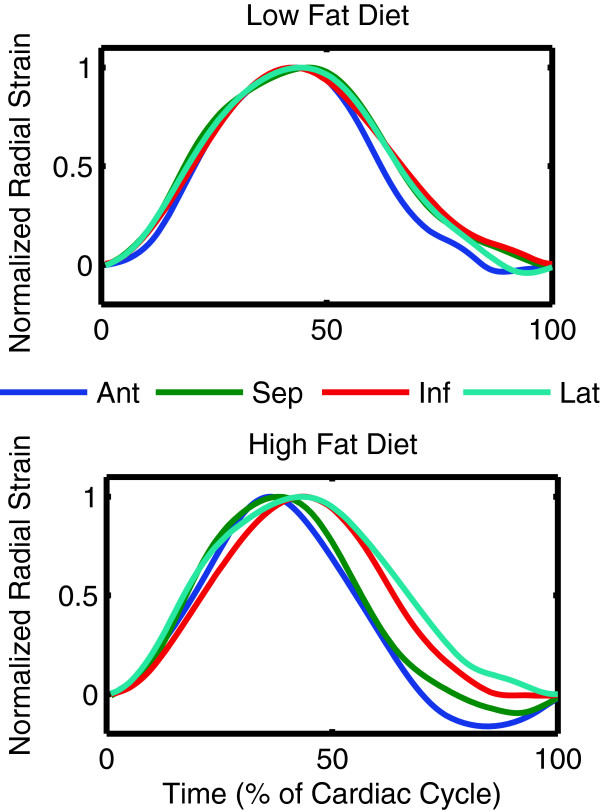
Average radial strain curves from control mice are highly uniform and synchronous whereas the radial strain curves from obese mice peak at different times in a dyssynchronous fashion.

The radial uniformity of strain index (RURE) was reduced from 0.95 ± 0.02 in the control mice to 0.91 ± 0.03 in the obese mice (p = 0.032) (Table 2, Figure 7). The circumferential uniformity of strain index (CURE) was not different in the obese mice (p = 0.151). Figure 8 shows the normalized average radial strain curves in the control and obese mice. All ventricular walls reach peak radial strain at the same time in the control mice. In the obese mice, the septum and anterior wall reach peak strain earlier than the inferior and lateral wall, which accounts for the reduced synchrony observed with the RURE index.

## Discussion

We used high-fat feeding to induce obesity in a mouse model to study the effects of obesity on the function of the heart using advanced measures of cardiac function (left ventricular strains, torsion and synchrony). Our major findings are that obesity is associated with: 1) left ventricular hypertrophy; 2) no change in ventricular volumes or ejection fraction; 3) reduced cardiac strains and torsion; 4) reduced synchrony of contraction in the left ventricle; and 5) a differential effect on strains in the myocardial layers of the ventricle, with the most severe depression of function in the subepicardium and little to no effect in the subendocardium.

These results are exciting as they show for the first time that mouse models of diet-induced obesity have changes in the heart that are similar to what has been reported in human studies: hypertrophy and reduced strains, torsion and synchrony in the setting of normal ejection fraction and normal ventricular volumes [27,28,38]. This work also highlights the growing importance of quantifying advanced measures of cardiac function in addition to the traditional measures of ventricular volumes and ejection fraction. On the basis of the traditional measures, there was no effect of obesity in this study; whereas the advanced measures clearly show a detrimental effect of obesity. These advanced measures may even be superior predictors of mortality in patients with cardiovascular disease [25,26] and have even shown abnormalities that are predictive of death in the general population, which further underscores their growing clinical value [39].

Identifying a precise mechanism underlying the effects of obesity on the heart is challenging. For example, mice with diet-induced obesity have mildly impaired glucose tolerance [40] but normal fasting blood glucose levels [41], suggesting these mice are not frankly diabetic. However, they do have chronically increased inflammatory cytokines [41] and an approximately 15 mmHg increase in systolic blood pressure [42]. Future studies may focus on isolating the different components of metabolic syndrome and their independent contributions to the observed remodelling (hypertrophy) and cardiac dysfunction we observed. It is important to note that obesity is associated with a wide range of other cardiovascular changes such as arterial stiffness [43] and changes in myocardial energetics [44], so it will be challenging to isolate all of these effects. Importantly, this isolation may be simpler to achieve in mouse models than in human studies; thus, the similarity of our findings in mice to human studies motivates these future mechanistic animal studies.

### Left ventricular dyssynchrony and obesity

To our knowledge, this is the first study to report dyssynchrony in the setting of diet-induced obesity in mice. This is a significant finding, since dyssynchrony has been reported in human obesity [38] and is known to lead to adverse remodeling which further exacerbates cardiac dysfunction [45]. For example, dyssynchrony creates regional differences in the amount of work an individual cardiac muscle fiber performs [46]. Early-activated regions contract against a lower pressure while late-activated regions contract against a higher pressure. This ultimately leads to hypertrophy in late-activated regions, which exacerbates the left ventricular dysfunction [47] and also worsens the dyssynchrony [48]. Thus, dyssynchrony “begets” dyssynchrony in a vicious cycle which leads to progressive worsening of cardiac function. Dyssynchrony could therefore play a primary role in the progression of cardiac dysfunction and failure in obesity. The dyssynchrony may be mediated through fibrosis of the conduction system, since obesity has already been shown to lead to cardiac fibrosis in previous studies [7]. Another potential mechanism could be a reduction in gap junction proteins in obese mice leading to dyssynchrony [49], but future studies will need to investigate these hypotheses.

Our results showed dyssynchrony according to the RURE index but not the CURE index. This could be due to the fact that the pathology underlying the dyssynchrony is not a typical conduction block but a more diffuse process such as changes in gap junctions which may affect the spread of conduction differently in the radial versus circumferential directions. However, we are confident that the observed dyssynchrony was a real phenomenon which can be clearly seen in Figure 8.

### Transmural heterogeneity in the effect of obesity on left ventricular strains

Our data show a heterogeneous effect of obesity on cardiac strains, with significant deleterious effects on the subepicardium, and relative preservation of function in the subendocardium. One potential mechanism for the heterogeneous effect may be paracrine inflammatory signaling from adjacent epicardial fat. An alternate explanation could be that myocytes in the subepicardium versus subendocardium have different mechanical function [50] and therefore respond differently to disease states. For example, the response to diseases such as heart failure [51] and myocardial infarction [52] have been shown to exhibit similar transmural heterogeneity. Future studies will need to investigate these hypotheses which have important implications for treatment and prognosis.

The left ventricular mass to end-diastolic volume ratio was increased in the obese mice, consistent with concentric hypertrophy. Concentric hypertrophy alters the loading conditions in the myocardium (wall stress) which can affect strains. Therefore, the remodeling in the hearts of the obese mice could in part explain some of the observed dysfunction. This is consistent with the significant correlations we found between strains and the mass to volume ratio (Table 3). However, there is evidence showing that myocyte contractile function is reduced in obesity, where the effect of loading is removed [53]. Thus, it is likely a combination of myocyte dysfunction and altered loading conditions due to the remodeling which contribute to the cardiac dysfunction in the obese mice.

### Study limitations

We used high-fat feeding to induce obesity in mice. The diet may have had an unanticipated acute effect on cardiac function, which our study did not consider. In fact, a recent study in humans showed that infusion of triglycerides acutely *improved* cardiac function [54]. While this acute improvement in cardiac function could not explain the differences we saw in our study, future studies should consider removing the high-fat diet for several days prior to CMR in order to minimize these potential acute effects.

We utilized 2-dimensional image slices to study 3-dimensional cardiac deformation. While 3-dimensional acquisition techniques for imaging deformation in the mouse heart have recently been developed [55], these techniques are more susceptible to off-resonance effects and in general more difficult to implement. Obese mice are particularly difficult to maintain stably under isoflurane anesthesia due to its high fat solubility, which makes multiple, shorter 2-dimensional image acquisitions more favorable than a longer, 3-dimensional acquisition. Moreover, the 2-dimensional imaging in our study was adequate to document significant differences in function between the obese and control mice.

We did not utilize a fat suppression technique during image acquisition. Future studies in obese mice may benefit from fat suppression to improve image quality. However, the overall quality of the images was good in both the obese and control mice (Figure 1).

Unfortunately, the quantification of torsion is not standardized in the literature. Published studies vary in both the slice selection to define the angular gradient and the normalization of the angular gradient to define torsion. We chose to normalize by the long-axis length of the ventricle, since there is evidence that this makes torsion values equivalent across mice and humans [35]. Of the studies which used similar methodologies, the reported torsion values of 0.28 – 3.15 degrees/mm are comparable to the value we obtained (0.88 degrees/mm).

## Conclusions

Diet-induced obesity leads to a reduction in cardiac function in mice as evidenced by reductions in left ventricular strains and torsion. Reductions in cardiac strain are mostly limited to the subepicardial layer of the left ventricle, with relative preservation of function in the subendocardium. Diet-induced obesity also leads to hypertrophy and reduced synchrony of contraction in the left ventricle of the heart. These findings help to further our understanding of the link between obesity and increased cardiovascular mortality, and also highlight important similarities between cardiac changes observed in obese mouse models and humans with obesity.

## Abbreviations

CMR: Cardiovascular magnetic resonance; CURE: Circumferential uniformity ratio estimate; DENSE: Displacement encoding with stimulated echoes; RURE: Radial uniformity ratio estimate.

## Competing interests

Dr. Epstein receives research support from Siemens.

## Authors’ contributions

SK completed the data analysis and drafted the manuscript. CH helped with data analysis and interpretation and drafting of the manuscript. CB and AM assisted with data collection and analysis and preparation of the manuscript. DP oversaw the CMR acquisition and also helped with data analysis and interpretation. LC participated in the study design and coordination, interpretation of results and drafting of the manuscript. FE developed the acquisition protocols and participated in the study design, interpretation of results and drafting of the manuscript. BF conceived the study, participated in its design and coordination, oversaw data acquisition and analysis, and helped to draft the manuscript. All authors read and approved the final manuscript.
